# TMEM100, a regulator of TRPV1-TRPA1 interaction, contributes to temporomandibular disorder pain

**DOI:** 10.3389/fnmol.2023.1160206

**Published:** 2023-03-23

**Authors:** Peng Wang, Qiaojuan Zhang, Fabiana C. Dias, Abbie Suttle, Xinzhong Dong, Yong Chen

**Affiliations:** ^1^Department of Neurology, Duke University, Durham, NC, United States; ^2^Solomon H. Snyder Department of Neuroscience, Johns Hopkins University School of Medicine, Baltimore, MD, United States; ^3^Center for Translational Pain Medicine, Department of Anesthesiology, Duke University, Durham, NC, United States; ^4^Department of Pathology, Duke University, Durham, NC, United States

**Keywords:** TMEM100, TRPA1, TRPV1, temporomandibular joint, masseter muscle, bite force, trigeminal ganglion sensory neurons, calcium signal

## Abstract

There is an unmet need to identify new therapeutic targets for temporomandibular disorder (TMD) pain because current treatments are limited and unsatisfactory. TMEM100, a two-transmembrane protein, was recently identified as a regulator to weaken the TRPA1-TRPV1 physical association, resulting in disinhibition of TRPA1 activity in sensory neurons. Recent studies have also shown that *Tmem100*, *Trpa1*, and *Trpv1* mRNAs were upregulated in trigeminal ganglion (TG) after inflammation of the temporomandibular joint (TMJ) associated tissues. These findings raise a critical question regarding whether TMEM100 in TG neurons is involved in TMD pain *via* regulating the TRPA1-TRPV1 functional interaction. Here, using two mouse models of TMD pain induced by TMJ inflammation or masseter muscle injury, we found that global knockout or systemic inhibition of TRPA1 and TRPV1 attenuated pain. In line with their increased genes, mice exhibited significant upregulation of TMEM100, TRPA1, and TRPV1 at the protein levels in TG neurons after TMD pain. Importantly, TMEM100 co-expressed with TRPA1 and TRPV1 in TG neurons-innervating the TMJ and masseter muscle and their co-expression was increased after TMD pain. Moreover, the enhanced activity of TRPA1 in TG neurons evoked by TMJ inflammation or masseter muscle injury was suppressed by inhibition of TMEM100. Selective deletion of *Tmem100* in TG neurons or local administration of TMEM100 inhibitor into the TMJ or masseter muscle attenuated TMD pain. Together, these results suggest that TMEM100 in TG neurons contributes to TMD pain by regulating TRPA1 activity within the TRPA1-TRPV1 complex. TMEM100 therefore represents a potential novel target-of-interest for TMD pain.

## Introduction

Temporomandibular disorders (TMD) include a group of painful conditions that involve the temporomandibular joint (TMJ), masseter muscles, and connective tissues (Scrivani et al., 2008). Mastication is fundamentally relevant for vertebrate nourishment. In cases of tissue inflammation or injury to the TMJ and masseter muscles, mastication becomes painful (Svensson et al., 1998; Scrivani et al., 2008; Iodice et al., 2013). Patients experience TMD pain which negatively impacts their overall quality of life. Current pharmacotherapies for TMD pain have limited efficacy and side effects (Andre et al., 2022). A deeper understanding of TMD pain mechanisms and identifying new targets for TMD pain are therefore warranted for developing more effective treatments.

TMD pain critically depends on trigeminal ganglion (TG) sensory neurons that transmit nociceptive signals from the peripheral tissues to the central nervous system. Detection of pain signals by these cells relies on specific transduction machinery. Key components thereof belong to TRP ion channels (Julius, 2013). TRPA1 and TRPV1, two cardinal pain-TRPs, have been critically implicated in both acute and chronic pain and represent possible bona-fide targets for the development of rationally-guided analgesics (Julius, 2013). However, their respective roles in TMD pain remain largely elusive. In addition, previous studies have suggested that TRPA1 and TRPV1 can form a complex of channel heteromers and functionally interact in sensory neurons (Salas et al., 2009; Akopian, 2011; Spahn et al., 2014). Yet, it is undetermined whether the interplay of TRPA1-TRPV1 in sensory neurons of the trigeminal system contributes to TMD pain.

TMEM100, a two-transmembrane protein, was recently identified as an adaptor to regulate the physical and functional interaction of TRPA1-TRPV1 in sensory neurons (Weng et al., 2015). TMEM100 weakens TRPA1-TRPV1 physical association, which results in disinhibition of TRPA1 activity. Conversely, when TMEM100 is absent, TRPV1 forms a tight complex with TRPA1 that greatly suppresses the activity of TRPA1 (Weng et al., 2015). These findings point toward TMEM100 as a potential new target for mitigating pathologic pain, a concept supported by available data on the loss-of-function of TMEM100. For instance, conditional knockout (cKO) of *Tmem100* in dorsal root ganglion (DRG) neurons or subcutaneous injection of TMEM100 inhibitor into the hindpaw blunted mechanical hyperalgesia caused by complete Freund’s adjuvant (CFA) in mice (Weng et al., 2015). While these data suggest that disabling TMEM100 may provide a novel strategy to combat DRG-mediated pain, contributions of TMEM100 to TG-mediated TMD pain await experimental clarification. TMD etiologies are attributable to anatomically and functionally unique target tissues (Piette, 1993; Detamore and Athanasiou, 2003). In addition, TMD pain not only has a strikingly different subjective quality when compared to DRG-mediated pain, but also different pharmacological responsiveness (Detamore and Athanasiou, 2003; Cairns, 2010; Andre et al., 2022; Tran et al., 2022).

Here, using two mouse models of chronic TMD pain, TMJ inflammation and masseter muscle injury as we previously described (Chen et al., 2013; Suttle et al., 2022), we sought to investigate whether: (1) genetic knockout or pharmacological inhibition of TRPA1 and TRPV1 attenuates TMD pain; (2) TMEM100 co-expresses with TRPA1 and TRPV1 in TG neurons and their co-expression is increased in TG neurons-innervating the TMJ and masseter muscle; (3) inhibition of TMEM100 suppresses the activity of TRPA1 in TG neurons; and (4) cKO of *Tmem100* in TG neurons or local injection of TMEM100 inhibitor into the TMJ and masseter muscle reduces TMD pain.

## Materials and methods

### Animals

*Trpa1* and *Trpv1* KO mice were from the Jackson Laboratory. *Tmem100* cKO (Advillin-cre^ER^::Tmem100^fl/fl^) mice were provided by Dr. Xinzhong Dong (Weng et al., 2015). Adv-Cre mice express tamoxifen-inducible Cre recombinase specifically in ~98% of TG/DRG neurons (Hasegawa et al., 2007). Deletion of *Tmem100* was induced *via* daily intraperitoneal (i.p.) injection of tamoxifen (75 mg/kg) for 5 days. Pirt-GCaMP3 mice expressing the genetically-encoded Ca^2+^ indicator GCaMP3 in >96% of DRG/TG neurons (Kim et al., 2014) were used for Ca^2+^-imaging. Male *KO* and cKO and their respective control mice (background: C57bl/6) were used at 2.5–3.5 months of age. Animals were housed in climate-controlled rooms on a 12/12 h light/dark cycle with water and food available *ad libitum*. Animal protocol was approved by Duke University- Institutional Animal Care and Use Committee (IACUC).

### Induction of TMJ inflammation and masseter muscle injury

TMD pain was induced in mice following studies (Guo et al., 2010; Chen et al., 2013; Bai et al., 2019; Suttle et al., 2022). For TMJ inflammation, mice were injected with 10 μl of complete Freund’s adjuvant (CFA, 5 mg/mL; Chondrex) into the joint. Controls received incomplete Freund’s adjuvant (IFA). For masseter muscle injury, ligation of the tendon of the anterior superficial part of masseter muscle (TASM) was conducted. The TASM was freed from surrounding connective tissues and the tendon was tied with two 6.0-chromic gut ligatures at 1.5 mm-apart during anesthesia with ketamine/xylazine (i.p. 80 mg/8 mg/kg, Sigma-Aldrich). Controls received the same procedure but the tendon was not ligated.

### TMD pain behavioral test

Bite force test was used to measure TMD masticatory pain as we previously described (Chen et al., 2013; Suttle et al., 2022). When bite transducer was slowly moved towards the mouse, a bite was invariably elicited. The voltage output during each bite was recorded using Labview 8.0 (National Instruments). The voltage of each bite was determined and converted into force (newton) based on the regression equation derived from calibration. Each animal was tested 3–5 times per time point and the values were averaged. The interval between two trials was >1 min. Mice were randomly assigned to treatment groups. The experimenter was blinded to the treatment conditions and genotypes. Although the baseline values for bite force were not statistically different between groups or genotypes, there were variations (12.84 to 16.13 newtons). To statistically analyze the data in a more objective way, bite force values after treatment were normalized to the baseline for each group and % of bite force changes were compared.

### Chemical injections

**Figure 1:**
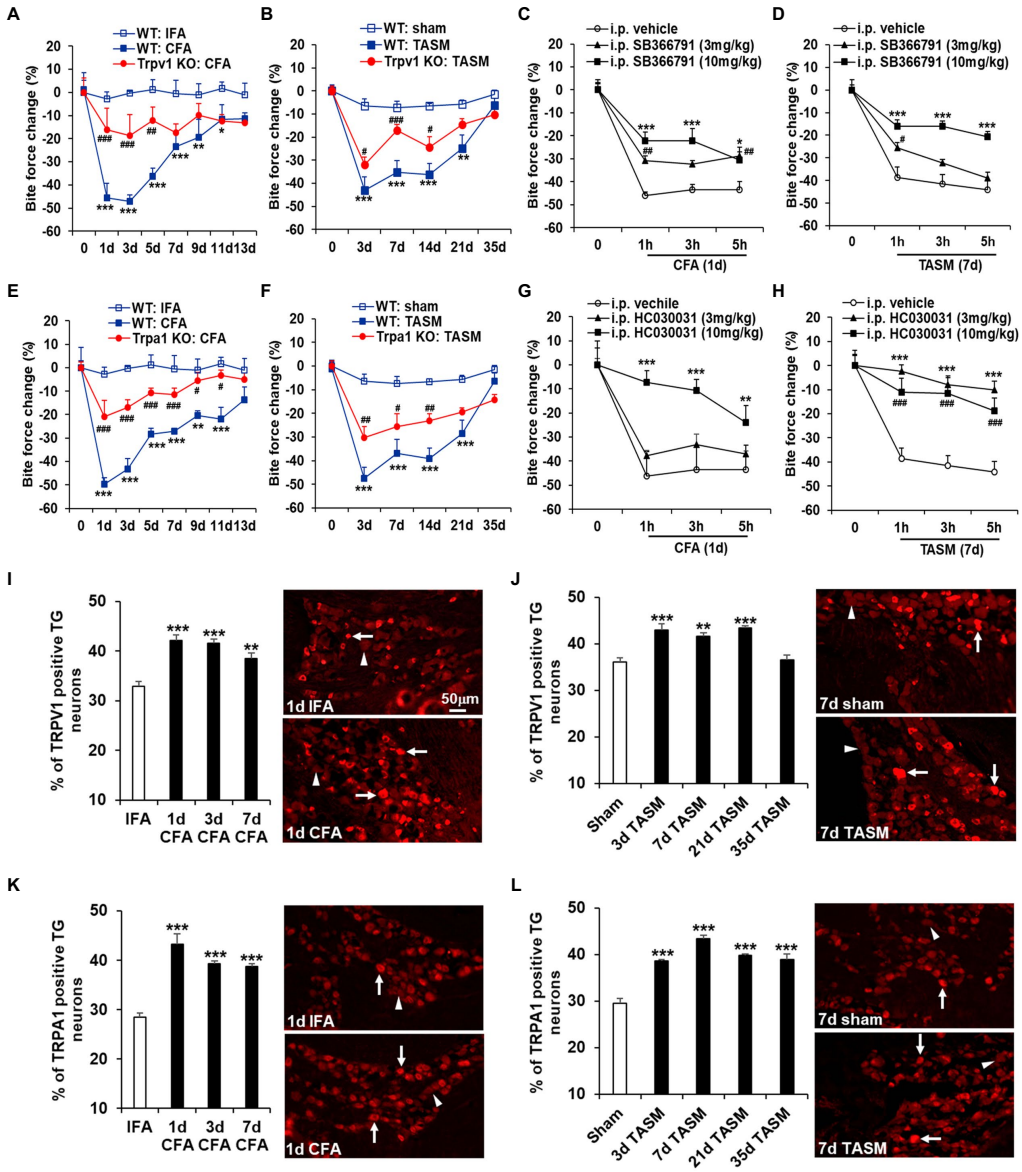
Global KO or systemic inhibition of TRPA1 and TRPV1 attenuates TMD pain. **(A,B,E,F)** TMJ inflammation or masseter muscle injury resulted in a long-lasting substantial reduction of bite force, which was attenuated by knocking out *Trpa1* or *Trpv1*. *p < 0.05, **p < 0.01, and ***p < 0.001 vs. WT: IFA or WT: sham; ^#^*p* < 0.05, ^##^*p* < 0.01, and ^###^*p* < 0.001 vs. WT: CFA or WT: TASM. Two-way ANOVA followed by Dunnett’s *post-hoc* test. *N* = 5–8 mice/group. (**C,D,G,H**) Single i.p. injection of the TRPV1 selective inhibitor SB366791 or TRPA1 selective inhibitor HC030031 at 3 mg/kg and 10 mg/kg significantly blunted the reduction of bite force caused by TMJ inflammation or masseter muscle injury except that HC030031 at 3 mg/kg had no significant impact on bite force reduction in TMJ inflammation model. *p < 0.05, **p < 0.01, ***p < 0.001, ^#^*p* < 0.05, ^##^*p* < 0.01, and ^###^*p* < 0.001 vs. i.p. vehicle (5% DMSO). Two-way ANOVA followed by Dunnett’s *post-hoc* test. *N* = 4–6 mice/group. Note: ‘0’ on x-axis represents baseline (before CFA or TASM). Exemplary changes of recorded bite force signal for WT: IFA, WT: CFA, and *Trpv1*-KO: CFA groups was shown in Supplementary Figure 1. (**I–L**) Temporal course of upregulation of TRPV1 and TRPA1 in TG neurons after TMJ inflammation or masseter muscle injury. (**I,J**) and (**K,L**) show increased percentage of TRPV1 and TRPA1 immunoreactive TG neurons after TMJ inflammation or masseter muscle injury, respectively. **p < 0.01 and ***p < 0.001 vs. IFA (1d) or sham (7d). One-way ANOVA followed by Dunnett’s *post-hoc* test. *N* = 4–5 mice/group. Arrows and arrowheads in images represent immunoreactive positive and negative neurons, respectively. TRPV1 and TRPA1 antibodies’ specificity was validated in TG sections from KO mice (Supplementary Figure 2).

To determine the effect of systemic inhibition of TRPA1 or TRPV1 on established TMD pain, mice were i.p. administered of the TRPA1 selective inhibitor HC030031 (Eid et al., 2008) or TRPV1 selective inhibitor SB366791 (Gunthorpe et al., 2004) (Sigma-Aldrich) on day 1 CFA or day 7 TASM, respectively, when TMD pain was the most prominent (see Figure 1). To test the local inhibitory effect of TMEM100, 10 μl of TMEM100 inhibitor T100-Mut (21stCenturyBio) was bilaterally, intraarticularly (i.a.) injected into the TMJs for CFA and intramuscularly (i.m.) injected into the masseter muscle for TASM models, respectively. T100-mut is a Tmem100-mutant-derived peptide which can selectively inhibit TRPA1 activity in TRPA1-TRPV1 complex (Weng et al., 2015). Control animals received 5% DMSO or normal saline.

To track TMJ or masseter muscle innervation by the TG neurons, mice were injected with 2 μl of neural tracer fast blue (FB, 2% aqueous solution; Polysciences) into the TMJ or masseter muscle 15 min before CFA/IFA and TASM/sham.

### Immunohistochemistry and quantitative analysis

Mouse TGs were dissected and sectioned at 12 μm. TG sections were blocked with 5% normal donkey serum (Jackson-ImmunoResearch) and incubated overnight with primary antibodies: guinea pig anti-TRPV1 (1:800, Neuromics), rabbit anti-TRPA1 (1:500, Aviva-Systems-Biology), sheep anti-TRPA1 (1:300, LifeSpan-Biosciences), and rabbit anti-TMEM100 (1:600, EMD Millipore). Immunodetection was accomplished with secondary antibodies (AlexaFluor-647, −594 and − 488; 1:600; Invitrogen) and cover-slipped with Vectashield (Vector). Images were acquired using BX61-Olympus upright microscope or Zeiss-780 confocal microscope. 4–6 sections/TG were analyzed. TG neurons were identified by morphology. Using ImageJ software (NIH), the cutoff density threshold was determined by averaging the density of three neurons/section that were judged to be minimally positive. All neurons for which the mean density exceeded the threshold of 25% were counted as positive.

### Ca^2+^-imaging of TG neurons

Following our method (Chen et al., 2021), *ex-vivo* Ca^2+^-imaging of TG explants was performed to visualize neuronal activity. TGs were dissected from Pirt-GCaMP3 mice and equilibrated in artificial cerebrospinal fluid (ACSF) bubbled with 95% O_2_/5% CO_2_ at room temperature. After 15 min, explants were placed in a dish with 2 ml of preoxygenated ACSF and imaged using Zeiss-780 upright confocal microscope with 20x water immersion objective at the 488-nm wavelength. TGs were stimulated with TRPA1 agonist JT010 (Takaya et al., 2015) (Sigma-Aldrich) at 100 nM. To examine whether inhibition of TMEM100 attenuates the JT010-induced Ca^2+^ signal, explants were pretreated with T-100 Mut at 200 nM during the 15 min sample equilibration. Ca^2+^ fluorescence intensity of each neuron before (baseline) and after (response) chemical stimulation was determined using ImageJ. The percentage of responding neurons was analyzed.

### Statistical analysis

Data were expressed as mean ± SEM. Two-tail *t*-test, one-way ANOVA or two-way ANOVA followed by Dunnett’s *post-hoc* test by Graphpad Prism 6 was used for groups comparison. Experimental “N” as used was based on a power analysis of our previous relevant studies involving bite force test, immunohistochemistry, and Ca^2+^-imaging (Chen et al., 2013, 2021; Suttle et al., 2022). *p* < 0.05 was considered statistically significant.

## Results

### Knockout or inhibition of Trpa1 and Trpv1 attenuates TMD pain

Whereas TMD has multifactorial etiologies (Ohrbach et al., 2011; Greenspan et al., 2013; Schiffman et al., 2014), a significant subgroup of patients suffer joint inflammation and/or masseter muscle injury (Stohler, 1999; Atsu and Ayhan-Ardic, 2006; Schiffman et al., 2014). To mimic these conditions in mice, we induced TMJ inflammation by injecting CFA into the joint and masseter muscle injury by ligating the TASM following our studies (Chen et al., 2013; Suttle et al., 2022). Bite force measurement, as a clinically relevant read-out, was used to assess masticatory pain of TMD (Chen et al., 2013). Both models resulted in long-lasting masticatory pain, as indicated by a substantial reduction of bite force from day 1 to 11 after CFA and from day 3 to 21 after TASM (Figure 1). KO of *Trpa1* or *Trpv1* significantly suppressed the reduction of bite force in both models (Figures 1A,B,E,F). In line with the results of knockout, systemic blockade of TRPA1 or TRPV1 by i.p. injection of HC030031 and SB366791, respectively, at 3 mg/kg and 10 mg/kg significantly blunted the reduction of bite force (Figures 1C,D,G,H). These data suggest that TRPA1 and TRPV1 are required for TMD pain.

### TRPA1 and TRPV1 expressions in TG neurons are increased after TMD pain

We have previously demonstrated that *Trpa1* and *Trpv1* mRNAs in TGs were increased after TMJ inflammation (Chen et al., 2013). Here, we extended this finding and found that the percentage of TRPA1- and TRPV1-immunoreactive TG neurons was elevated after TMJ inflammation, as well as after masseter muscle injury (Figures 1I–L). These data suggest that TG neurons might be the crucial cellular site where TRPA1 and TRPV1 drive TMD pain.

### TMEM100 co-expresses with TRPA1 and TRPV1 in TG neurons and their co-expression is increased after TMD pain

**Figure 2:**
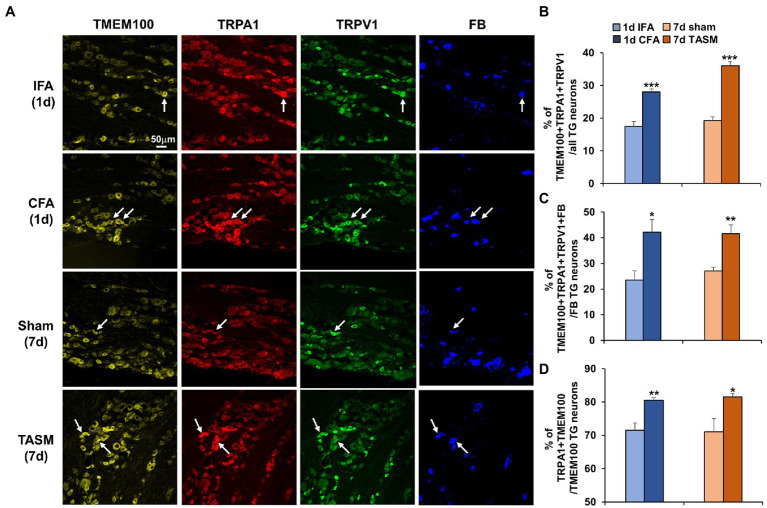
Co-expression of TMEM100 with TRPA1 and TRPV1 in TG neurons-innervating the TMJ and masseter muscle is increased after TMJ inflammation or masseter muscle injury. **(A)** Representative images show colocalization of TMEM100, TRPA1, TRPV1, and FB in TG neurons. Arrows in images represent colocalized neurons. **(B–D)** Quantitative analysis shows an increased percentage of TMEM100 + TRPA1 + TRPV1/total of TG neurons **(B)**, TMEM100 + TRPA1 + TRPV1 + FB/FB-labeled TG neurons **(C)**, and TRPA1 + TMEM100/TMEM100 positive neurons **(D)** 1 day after CFA or 7 days after TASM. *p < 0.05, **p < 0.01, and ***p < 0.001 vs. IFA or sham, two-tail *t* test. *N* = 4–5 mice/group. TRPV1 and TRPA1 antibodies’ specificity was validated in TG sections from KO mice (Supplementary Figure 2) and TMEM100 antibody specificity was previously validated (Yu et al., 2019).

Immunostaining demonstrated that TMEM100 co-expressed with TRPA1 and TRPV1 in TG neurons (Figure 2A). Interestingly, their colocalization was substantially increased after CFA or TASM (TMEM100 + TRPA1 + TRPV1/all TG neurons, Figure 2B). Further, their co-expression in TG neurons-innervating the TMJ or masseter muscle was also significantly increased after CFA or TASM (TMEM100 + TRPA1 + TRPV1 + FB/FB-labeled neurons, Figure 2C). Studies have shown that 45 and 96% of TRPA1 co-express TMEM100 in DRG neurons in mice (Weng et al., 2015) and rats (Yu et al., 2019), respectively, we found that ~71% of TRPA1 co-expressed with TMEM100 in TG neurons of control mice (Figure 2D). These discrepancies might be due to TG vs. DRG and mice vs. rats. Importantly, we found co-expression of TRPA1 with TMEM100 in TG neurons (TRPA1 + TMEM100/TMEM100) was significantly increased after CFA or TASM (Figure 2D).

### Inhibition of TMEM100 suppresses the enhanced activity of TRPA1 in TG neurons after TMD pain

**Figure 3:**
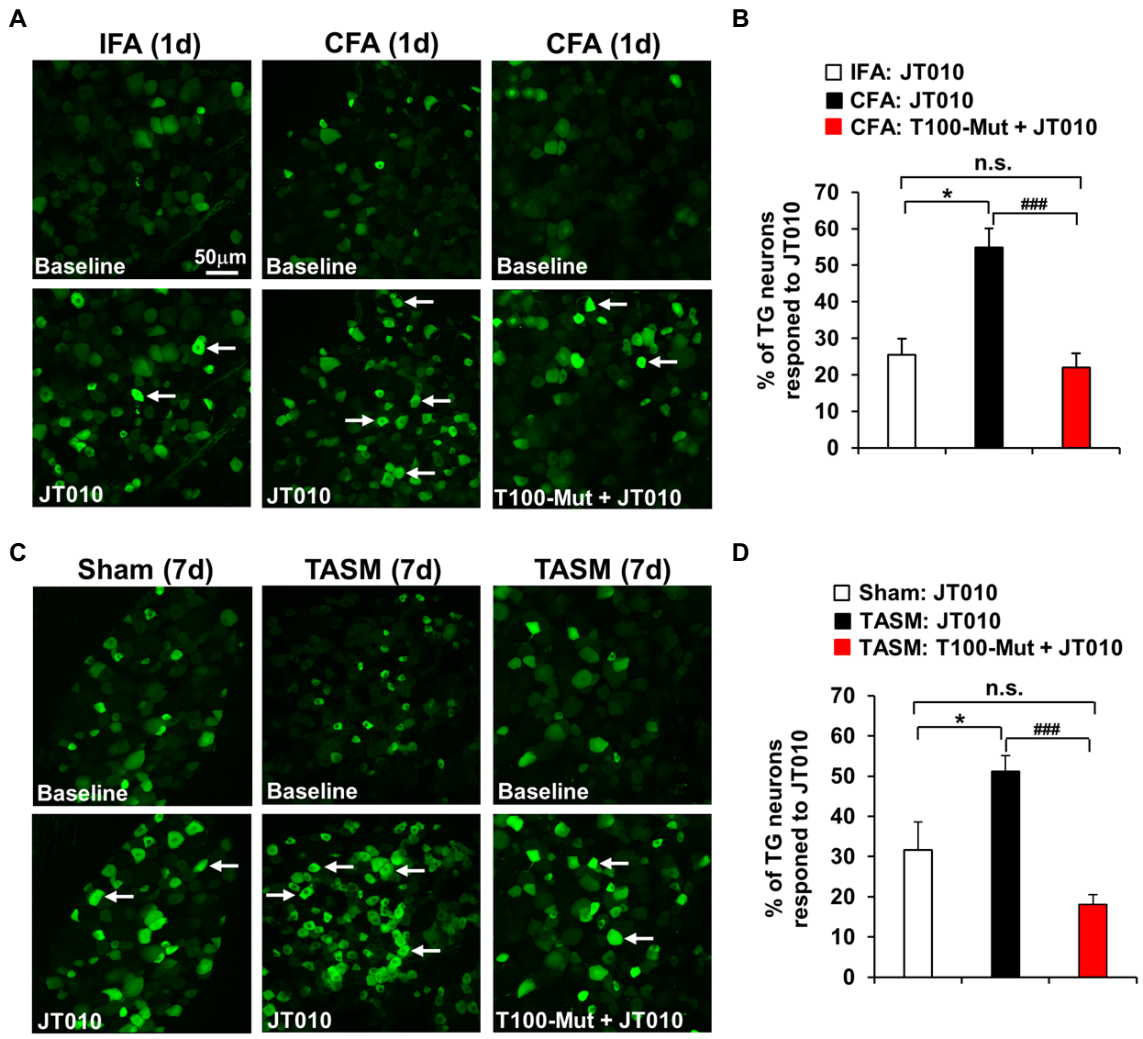
Inhibition of TMEM100 suppresses the enhanced neuronal activity of TRPA1 in TG after TMJ inflammation or masseter muscle injury. **(A,C)** Representative Ca^2+^-imaging of GCaMP3-expressing TG neurons in an *ex-vivo* preparation illustrates the increased Ca^2+^-signal in response to the TRPA1 selective agonist JT010 (100 nM) 1 day after CFA (**A**) or 7 days after TASM (**C**), which was reduced by pretreatment of the TMEM100 inhibitor T100-Mut (200 nM). **(B,D)** Quantitative analysis shows increased percentage of total TG neurons responding to JT010 after CFA **(B)** or TASM **(D)** was reduced by T100-Mut. *p < 0.05 and ^###^*p* < 0.001, one-way ANOVA followed by Dunnett’s *post-hoc* test. *N* = 4–8 mice/group. Arrows in images represent responder neurons.

Co-localization of TMEM100 with TRPA1 and TRPV1 in TG neurons possibly provides a cellular basis for TMEM100 to regulate TRPA1 activity in TRPV1-TRPA1 complex. We then examined whether TRPA1 activity in TG neurons is enhanced after TMD pain, more importantly, whether this enhancement can be suppressed when inhibiting TMEM100. *Ex vivo* Ca^2+^-imaging demonstrated an increased percentage of neurons responding to TRPA1 agonist JT010 in organotypic TGs prepared from mice received CFA or TASM (Figure 3). Importantly, this increase was conspicuously reduced by the TMEM100 inhibitor T100-Mut. Of note, T100-Mut did not abolish TRPA1 activity, possibly due to a fraction of TRPA1-expressing neurons which do not contain TMEM100 (Figure 2D). Nevertheless, these data suggest that TMEM100 regulates TRPA1 activity in TG neurons under TMD pain.

### cKO of Tmem100 in TG neurons or local administration of T100-Mut into the TMJ or masseter muscle attenuates TMD pain

**Figure 4:**
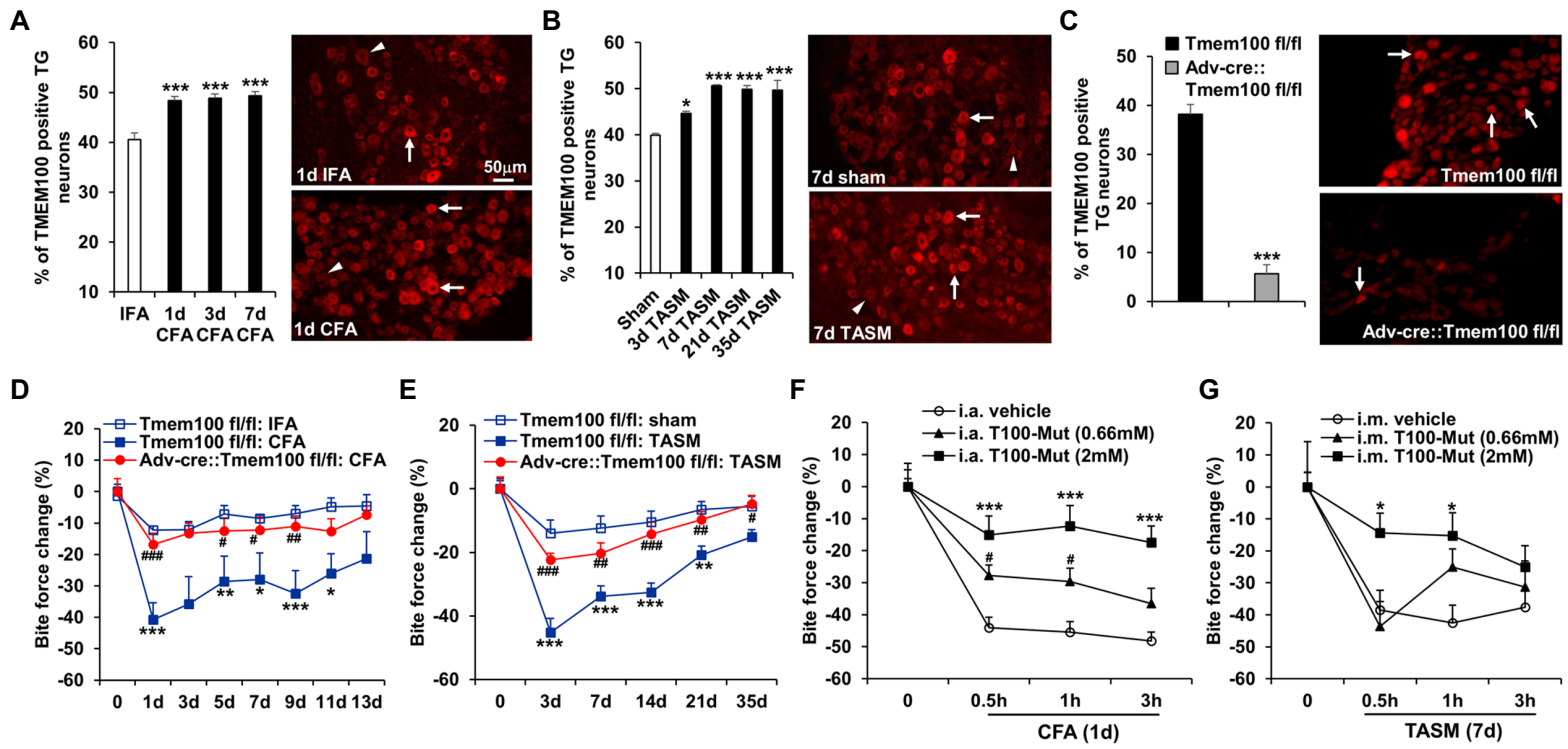
Conditional KO of *Tmem100* in TG neurons or local administration of TMEM100 inhibitor attenuates TMD pain. **(A,B)** TMJ inflammation **(A)** or masseter muscle injury **(B)** led to an increased expression of TMEM100 in TG neurons. *p < 0.05 and ***p < 0.001 vs. IFA or sham. One-way ANOVA followed by Dunnett’s *post-hoc* test. *N* = 4–5 mice/group. Arrows and arrowheads in images represent TMEM100 immunoreactive positive and negative neurons, respectively. **(C)** Depletion of TMEM100 in TG neurons in *Tmem100* cKO mice. Immunostaining shows dramatic reduction of TMEM100-immunoreactive TG neurons in Adv-cre::Tmem100^fl/fl^ mice after tamoxifen treatment. ***p < 0.001, two-tailed *t*-test, *n* = 3–4 mice/group. **(D,E)** cKO of *Tmem100* in TG neurons attenuates TMD pain after TMJ inflammation **(D)** or masseter muscle injury **(E)**. *p < 0.05, **p < 0.01, and ***p < 0.001 vs. Tmem100^fl/fl^: IFA or sham; ^#^*p* < 0.05, ^##^*p* < 0.01, and ^###^*p* < 0.001 vs. Tmem100^fl/fl^: CFA or TASM. Two-way ANOVA followed by Dunnett’s *post-hoc* test. *N* = 4–9 mice/group. **(F,G)** local injection of the TMEM100 inhibitor T100-Mut into the TMJ (i.a.) or masseter muscle (i.m.) at 0.66 mM and 2 mM suppressed the reduction of bite force after CFA **(F)** or TASM **(G)** except that T100-Mut had no effect on bite force reduction at 0.66 mM in TASM model. *p < 0.05, ***p < 0.001, and ^#^*p* < 0.05 vs. i.a. or i.m. vehicle (normal saline). Two-way ANOVA followed by Dunnett’s *post-hoc* test. *N* = 5–7 mice/group. Note: ‘0’ on x-axis represents baseline (before CFA or TASM).

It was reported that *Tmem100* mRNA in TG was upregulated after inflammation of masseter muscle (Chung et al., 2016). Consistently, we found TMEM100 expression in TG neurons is increased after TMJ inflammation or masseter muscle injury (Figures 4A,B). We next examined whether deletion of *Tmem100* in TG neurons reduces TMD pain. In Adv-Cre::Tmem100^fl/fl^ mice after tamoxifen treatment, we found TMEM100 expression in TG neurons was dramatically reduced (Figure 4C), validating the knockout efficiency. Behavioral assays revealed that cKO of *Tmem100* in TG neurons significantly suppressed the reduction of bite force in both models (Figures 4D,F). Considering co-expression of TMEM100, TRPA1, and TRPV1 in TG neurons-innervating the TMJ and masseter muscle is elevated after TMD pain (Figure 2), we next determined the effect of local inhibition of TMEM100 in these tissues on TMD pain. Interestingly, we observed that i.a. or i.m. injection of T100-Mut at 0.66 mM and 2 mM [10 μl, doses chosen based on Weng et al. (2015)] into the TMJ or masseter muscle for CFA and TASM models, respectively, attenuated the reduction of bite force (Figures 4E,G). These data suggest that sensory neuron-TMEM100 contributes to TMD pain.

## Discussion

Although TMEM100 has been demonstrated to modulate pain *via* regulating TRPA1-TRPV1 interaction at the DRG level (Weng et al., 2015), experimental evidence regarding whether TMEM100 controls orofacial pain at the TG level remains lacking. Here, we found that genetic knockout or pharmacological inhibition of TRPA1 and TRPV1 attenuated TG-mediated TMD pain. We further demonstrated that TMEM100 colocalized with TRPA1 and TRPV1 in TG neurons and their co-expression was increased in TG neurons-innervating the TMJ and masseter muscle after TMD pain. Importantly, inhibition of TMEM100 suppressed TRPA1 activity in TG neurons and cKO of *Tmem100* in TG neurons or local injection of TMEM100 inhibitor into the TMJ and masseter muscle attenuated TMD pain. These findings suggest that TMEM100 in TG neurons contributes to TMD pain through regulating TRPA1 activity in TRPA1-TRPV1 complex.

Therapeutic targets for TMD pain are desirable because current pharmacotherapeutics have limited efficacy and side effects (Cairns, 2010; Andre et al., 2022; Tran et al., 2022). TRPA1 and TRPV1 have been implicated in a variety of pain states, including trigeminally-mediated pain (Julius, 2013; Luo et al., 2021). For instance, TRPA1 and TRPV1 might be involved in migraine, headache and dental pain (Benemei et al., 2013; Luo et al., 2021), for which TMD shares significant co-morbidity (Ohrbach et al., 2011). In addition, TRPV1 has been detected in TG neurons-innervating the TMJ, masseter muscle, and synovial lining cells (Sato et al., 2005; Lund et al., 2010; Lindquist et al., 2021). Notably, we and others previously found that inflammation of TMJ-associated tissues upregulates *Trpa1* and *Trpv1* genes in TG (Chen et al., 2013; Chung et al., 2016). These findings raise an important question as to whether TRPA1 and TRPV1 are involved in TMD pain. Here, we found that TMJ inflammation as well as masseter muscle injury led to an upregulation of TRPA1 and TRPV1 in TG neurons at the protein levels. Importantly, bite force reduction was substantially attenuated by global KO or systemic inhibition of these ion channels, providing an important demonstration that TRPV1 and TRPA1 are essential for TMD pain. Interestingly, using bite force test, Wang et al. previously found that TRPV1 has a marginal effect and TRPA1 has no influence on pain induced by CFA injection into the masseter muscle (Wang et al., 2017, 2018). It is worth noting that bite force reduction induced by masseter myositis in these studies was of short duration, i.e., <3 days, which contrasts with pain-behavior duration of TMJ inflammation (>11 days) or masseter muscle injury (>21 days) in our study. Therefore, one possible explanation for these discrepant findings might be due to distinct mouse models.

TRPA1 and TRPV1 in sensory neurons can form a complex and functionally interact to modulate pathological pain (Salas et al., 2009; Akopian, 2011; Spahn et al., 2014; Weng et al., 2015). Interestingly, it was recently discovered that TMEM100 contributes to DRG-mediated pain *via* impairing TRPA1 function in TRPA1-TRPV1 complex (Weng et al., 2015). In the present study, several lines of evidence demonstrate that TMEM100 modulates TG-mediated TMD pain. First, TMEM100 co-expressed with TRPA1 and TRPV1 in TG neurons-innervating the TMJ and masseter muscle and their co-expression was elevated after TMD pain. These data suggest that TG neurons may provide a critical cellular locale whereby TMEM100 mediates TMD pain *via* governing the functional interaction of TRPA1-TRPV1. Second, to gain a mechanistic insight into the role of TMEM100 in TMD pain, our Ca^2+^ imaging assay showed that the percentage of TG neurons responsive to TRPA1 agonist JT010 was increased after TMJ inflammation or masseter muscle injury. Importantly, the enhanced activity of TRPA1 was suppressed when inhibiting TMEM100, suggesting a regulatory role of TMEM100 on TRPA1 function in trigeminal sensory system. Third, selective deletion of *Tmem100* in TG neurons or local injection of TMEM100 inhibitor into TG neurons-innervating TMJ and master muscle tissues significantly blunted TMD pain. These compelling data suggest that TMEM100 might form a multi-protein complex with TRPA1 and TRPV1 in TG neurons and mediate TMD pain through regulating TRPA1 activity within the complex. Although there are some distinct differences in pain processing mechanisms, anatomical specificities, and behavioral phenotypes between spinal and trigeminal systems under pathophysiological states (Luo et al., 2021), our findings support a novel concept that TMEM100 contributes to trigeminally-mediated pain. Future studies are needed to investigate whether TMEM100 is also involved in modulating other types of trigeminal pain, such as dental pain, migraine, and trigeminal neuralgia. Interestingly, a recent study reported that TMEM100 co-expresses with TRPA1 and TRPV1 in human odontoblasts, implicating a potential role of TMEM100 in dental pain (Liu et al., 2021). In addition, based on the data showing that 95 and 74% of TMEM100-positive sensory neurons express CGRP in mice (Weng et al., 2015) and rats (Yu et al., 2019), respectively, and in view of CGRP’s crucial role in migraine (Dussor, 2019), it would be interesting to investigate whether TMEM100 contributes to migraine *via* regulating CGRP levels.

There is a critical need for identifying novel targets for TMD pain and better understanding of its underlying mechanisms because TMD pain is inadequately managed. Our study for the first time demonstrates that TMEM100 contributes to TG-mediated TMD pain *via* a mechanism of action involving the regulatory effect on TRPA1 activity in TRPA1-TRPV1 complex. TRPA1 and TRPV1 are recognized as analgesic targets for development of effective therapies. However, clinical trials based on their inhibitors are delayed or stuck due to off-target thermoregulatory effects and blunting of normal noxious sensation (Moran, 2018). Thus, new modes of regulating the ion channel’s activity rather than inhibition of ion channel *per se* in pain transmission pathways represent promising alternative approaches to combat pain. Given that the TMEM100 inhibitor can attenuate TMD pain when it is locally administrated into TMJ or masseter muscle tissues, our study implicates that TMEM100 could serve as a peripheral-topical target for reducing TMD pain while avoiding potential side effects of systemic treatments.

## Data availability statement

The original contributions presented in the study are included in the article/supplementary material, further inquiries can be directed to the corresponding author/s.

## Author contributions

PW contributed to conception, design, data acquisition, analysis, interpretation, and critically revised the manuscript. QZ, FD, and AS contributed to data acquisition analysis, interpretation, and critically revised the manuscript. XD contributed to conception and critically revised the manuscript. YC contributed to conception, design, data acquisition, analysis, and interpretation, drafted and critically revised the manuscript. All authors gave final approval and agreed to be accountable for all aspects of the work.

## Funding

This research was supported by the National Institutes of Health R01DE027454 and R01DE027454-02S2 (YC) and R01GM087369 (XD).

## Acknowledgments

We thank Steven Shen’s technical support.

## Conflict of interest

The authors declare that the research was conducted in the absence of any commercial or financial relationships that could be construed as a potential conflict of interest.

## Publisher’s note

All claims expressed in this article are solely those of the authors and do not necessarily represent those of their affiliated organizations, or those of the publisher, the editors and the reviewers. Any product that may be evaluated in this article, or claim that may be made by its manufacturer, is not guaranteed or endorsed by the publisher.
