# Supplementary figures and images for: TMEM100, a regulator of TRPV1-TRPA1 interaction, contributes to temporomandibular disorder pain
Source: Front Mol Neurosci. 2023 Mar 23;16:1160206. doi: 10.3389/fnmol.2023.1160206 (PMC10077888; doi:10.3389/fnmol.2023.1160206)

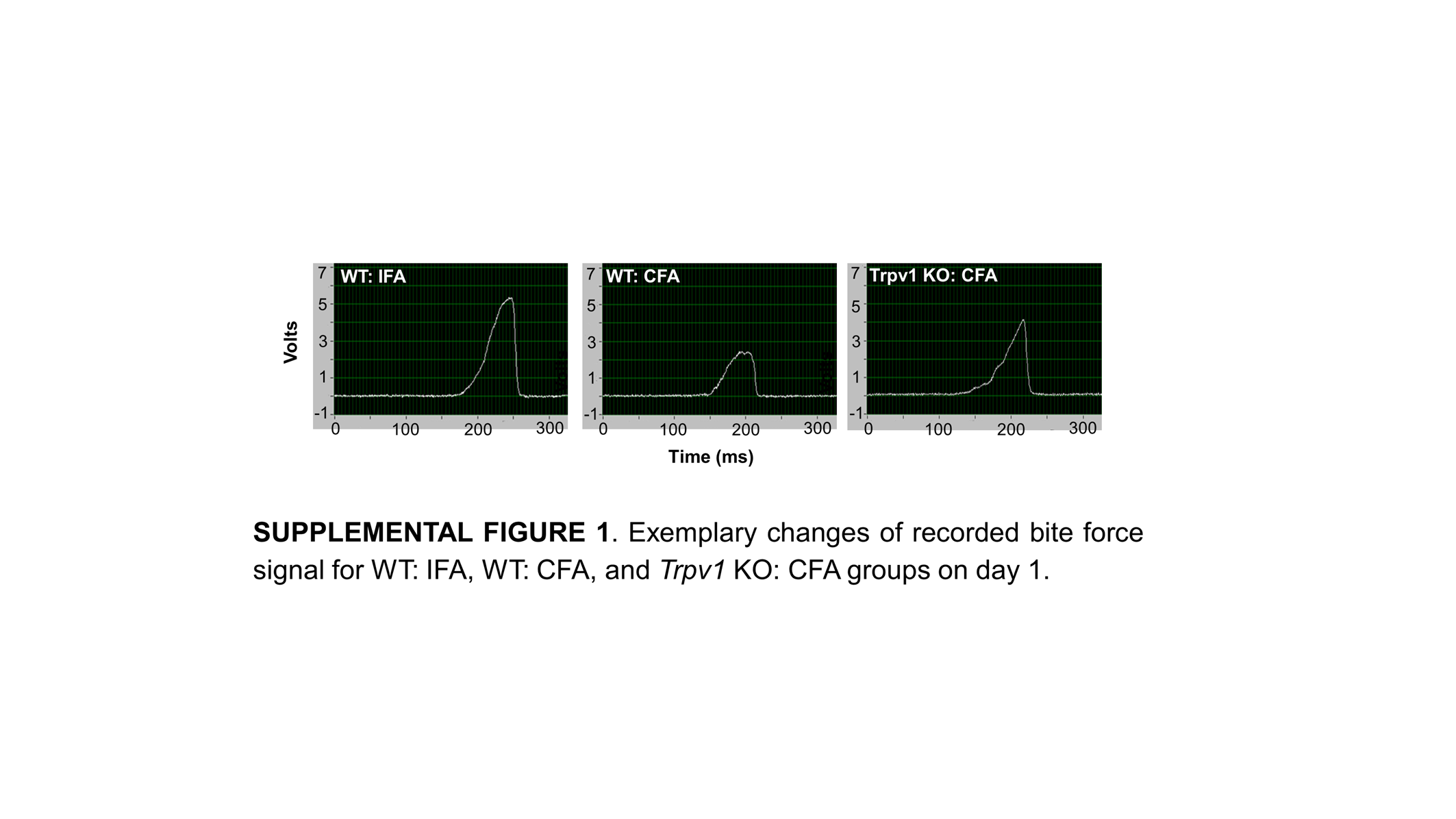

Supplement: Supplementary file 1 [file Image_1.TIF]

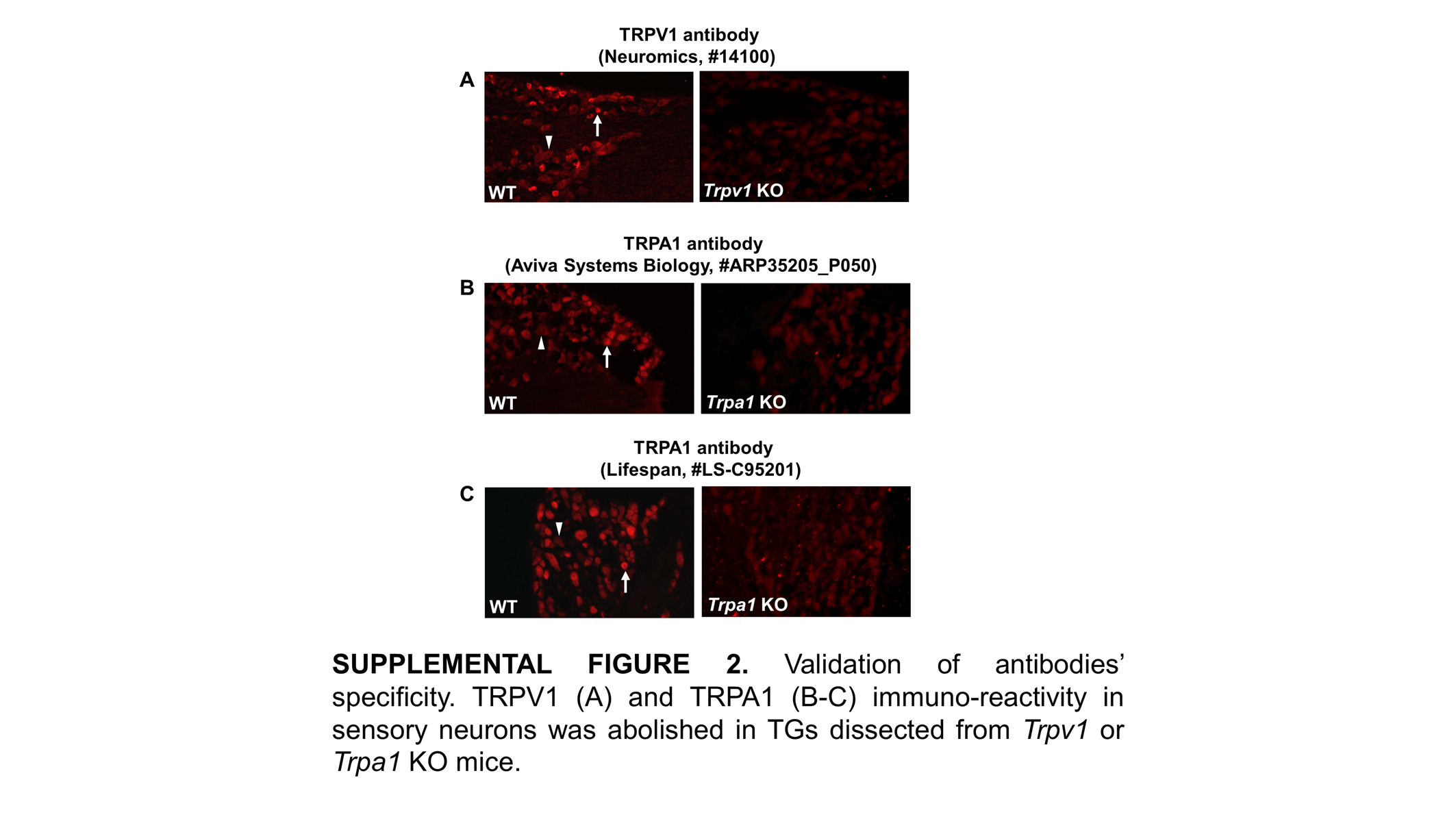

Supplement: Supplementary file 2 [file Image_2.TIF]
